# Canine Influenza—Its Symptoms and Treatment

**Published:** 1899-12

**Authors:** H. Karslake Tasker

**Affiliations:** Bexhill-On-Sea


### SELECTIONS.

### CANINE INFLUENZA—ITS SYMPTOMS AND TREATMENT.

By H. Karslake Tasker, M.R.C.V.S.,
BEXHILL-ON-SEA.

The various articles headed "Canine Influenza," "New Diseases in Dogs," "Gastro-enteritis," etc., which have appeared in the pages of the *Veterinary Record* of late, are, I am sure, most interesting, especially to veterinary surgeons who, like myself, have made a specialty of canine surgery and pathology. It strikes me very forcibly that there exists amongst members of our profession great diversity of opinion as to what this new disease amongst dogs really is. Although I do not claim to be an old practitioner, nor wish in any way to preach or try to teach my elders, I nevertheless venture to hope that a few remarks on this new disorder affecting dogs, based only upon continued hard work and observations made upon cases under my care, may not be considered out of place, and may even be worthy of the attention of the readers of the *Record*.

To study this disease properly I have considered it expedient in some cases to sit up with the dogs all night to notice the symptoms from the commencement of the disease to its finish.

My experience up to the present date leads me to pronounce at once that this new disease in dogs is nothing more nor less than "influenza" in its various forms and complications. Let me, therefore, give my reasons for this statement.

As far as I am aware influenza is liable to attack any animal. It seems even communicable from man to animals, but not so communicable from animals to man.

Whilst in Edinburgh, as a student, I turned my attention to studying the disease both in man and animals. I have noticed in houses in which the inhabitants were suffering from or recovering from influenza that an animal, such as a dog or a cat, and in some cases even the tame canary, would invariably take the disease last. Again, when a dog is brought to me showing marked signs of influenza in one of its forms, on inquiry I generally elicit the fact that someone in the house from which it comes has influenza or is recovering from it. This leads me to theorize that the period of incubation of the disease in dogs is longer than that in the human being. It gives room for the theory that influenza may be caused by the entrance of a microorganism into the system.

Beside being infectious from man to dogs the disease is also infectious or contagious from dog to dog. I will now give a brief review of the disease and its treatment.

Canine influenza might be defined as an epizoötic febrile disease, subject to various forms and complications, with, in most cases, marked prostration of strength, accompanied by inflammation of the nasal and conjunctival mucous membranes, often accompanied by an inflammation of the mucous membranes of the alimentary canal and other mucous membranes.

Forms. I have divided the disease into six different forms, so as to describe the symptoms more accurately, viz.: 1, catarrhal; 2, thoracic; 3, gastric; 4, gastro-enteric (or abdominal forms); 5, paralytic; 6, transitory.

*Catarrhal Form.* The first symptoms I have noticed previous to all the forms of canine influenza is that the animal's nose becomes dry and is hot to the touch. After two or three days the animal is usually affected with a peculiar cough. It is a loud, prolonged, snorting cough, something between a sneeze and cough—in fact, it sounds as if the animal was trying to cough and sneeze up a discharge from the nostrils and mouth at one and the same time—but there is, at this stage, *no discharge.* It is not an incessant cough, and may only occur from two to five times a day, but more especially in the evening, the animal otherwise appearing in the best of health. To this I give the name of the catarrhal form, as it so resembles an ordinary cold and is apt to be overlooked; now is the time to use a preventive treatment.

*Thoracic Form.* Next may be noticed a weakness of the eyes and a flow of tears down the cheek, or most often extra moisture at the inner canthus of the eyes. Then a clear discharge from the nostrils. After a few days the discharge from the eyes becomes whitish in color, and that from the nostrils also, the latter sticking round the openings of the nasal chambers. The dog at this stage suddenly refuses all food, and has little strength to move, only being able to walk a few yards, and then fall down exhausted. Here we have the "thoracic form," because in this stage we usually can detect inflammation of the larger bronchial tubes. These symptoms, up to this stage of prostration, take from seven to fourteen days to develop after the cough is first noticed, but the period is variable. On opening the mouth the mucous membranes are hot and red, as also are the conjunctivæ. The breathing is labored and low, exhausting fever present, the animal often making attempts to cough away the discharge. We will say, for example, that we are attending a dog at this stage of the disease—*the stage of prostration.* The animal will usually show aggravation of the symptoms at from 12.30 A. M. to 2.30 A. M.; therefore it is an important matter to stay up with the animal the first night, at least, and keep on with *constant feeding* and stimulants. If the case progresses favorably a slight improvement will be found on the second day; there will also be more discharge from the eyes and nose. About the third or fourth day the discharge will become yellowish in color; about the sixth day it will absolutely run from the nose, and then it again becomes whitish in color, but very little in amount, and stops altogether on the eighth, ninth or tenth day. When this occurs the cough either altogether stops or alters its character to an ordinary cough. The discharge from the eyes gets more abundant as the discharge from the nose gets less; it keeps on for about a day or two after that from the nose stops, and it then stops suddenly. During this time the animal under treatment gradually picks up its strength, and can be exercised after the fourth day; the appetite becomes better on the fourth or fifth day, but, of course, the patient is not out of danger for a week, as the least chill is apt to prove fatal. As a rule the animal recovers in fourteen days after the stage of prostration commences, but is left in a weak state for a week or two, having lost weight considerably during the progress of the disease. These two forms appear to me easy to bring to a successful termination if the animal is carefully treated, provided he does not die during the first three days, which seems the most uncertain time.

*The Gastric Form* occurs after the cough has been noticed for about two weeks. The animal is suddenly attacked with violent vomiting, and loses condition rapidly (owing to the inability of the stomach to retain food), bringing up at each vomit a lot of white or yellowish frothy matter. In this form there may be no discharge from the eyes or nose. The latter organs being very hot the animal lies about, as a rule, in cool places. This gastric form, if neglected, will, in all probability, lead on to the much-discussed gastro-enteric form of influenza, although, in many cases, the gastro-enteric form does not follow the gastric, but appears as a separate form of the disease. A dog may be noticed to be unwell one day, vomiting occasionally; next day he is a good deal worse, the vomit being streaked or colored with a darkish-brown material or streaked with blood; on the third day the dog usually dies. During this period diarrhœa is often a symptom, this being stained with the same material or with blood.

*Post-mortem Examination.* The post-mortem appearances in the gastro-enteric form of influenza are peculiar. The stomach *in situ* has the appearance of a chocolate-colored tumor; the peritoneal coat of the bowels may be inflamed throughout, inflamed in patches, or not show any signs of inflammation. On cutting open the bowels the entire length of the mucous coat is usually inflamed, and the bowels contain a gelatinous material, chocolate, dark red, or pink in color. This may be of a uniform color throughout the length of the bowels, or a patch of one color and then another. The stomach, when opened, shows that the inflammation has extended through all its coats; it contains this chocolate-colored gelatinous material, but it is darker than in the bowels. It would appear that the mucous coat of the stomach and intestines undergoes a catarrhal inflammation, succeeded by a gelatinous degeneration.

I cannot at all understand why this form of influenza should be looked upon as a "new disease." I am informed by doctors that influenza in human patients is now taking the gastro-enteric form, and why it should not also attack the dog in the same way I cannot comprehend. It is certainly a very fatal form of the disease if not taken in good time.

*Paralytic Form.* The paralytic form, by which I mean that state when, combined with any other forms of the disease, paralysis of both hind-legs sets in, and any attempt to hold up the animal results in the hind-legs falling under him or falling to the side. When this paralysis sets in the case is usually hopeless.

*Transitory Form.* I have named my last form of influenza the transitory form. In this the symptoms are very slight, and resemble, on a minor scale, any of the forms of influenza alluded to. They may pass off in a few days with careful treatment.

TREATMENT. I do not wish in any way to boast of my professional abilities, or medicines, etc., prepared or sold by me, but most of the cases of influenza that have been put under my care have recovered. If any animal survives when he is at first in a condition of great prostration for three or four days a recovery may be looked for with every hope, and certainly if the dog continually improves for a week, recovery is the rule, not the exception. I have had two cases linger on for from five to six weeks and then die, but I traced these to the non-attention of my rule of feeding and medicinal treatment at the commencement, consequently the animals were attacked with complications and died; being myself unwell at the time I had to trust to others to do the work, with this result.

*Catarrhal and Transitory Forms.* Directly the dog shows signs of the peculiar cough mentioned I dose him two or three times a day with a special influenza pill (made to my prescription by Messrs. Willows, Francis & Butler, 40 Aldersgate Street) until the cough ceases. These pills, if given in time, appear to me to ward off attacks of influenza, my own dog being quite free from it, although he is frequently about with those affected with it. I dosed him for five days directly he commenced to cough, and now give him the pills occasionally.

I give the manufacturers full leave to supply these pills to members of the profession for trial, and can only hope that they may find their use as beneficial in the treatment of the disease as I have. I do not feel obliged in anyway to write out the prescription at present, but simply to state that the combination of the ingredients of the pills has been thought out with hints from a medical friend.

*Thoracic Form.* When a dog is brought to me with symptoms such as I have described under this head, in a state of absolute prostration, I commence with him at once, as follows: First, give a sniff of amyl nitras, then bathe the discharge well away from the nostrils; insert an eyelid retractor, and clean out all the discharge from between the lower eyelids and eye, where it will be found to have partially coagulated in a long mass, using a mixture for this purpose of two ounces hamamelis

Virginica and six ounces of warm water. Injecting up each nostril some hamamelis Virginica dest., pure (Burrows & Wellcome), next I give one drachm of ammoniated tincture of quinine and one drachm of brandy, in one and one-half ounces of cold milk; place the dog in a comfortable position and leave him quiet for half an hour; then give one egg beaten up in a half-pint of milk (or kreochyle). This treatment I repeat every two or three hours for the first day and the greater part of the night, but not always giving the quinine, using about two ounces altogether the first day. By the next morning the animal, unless it is a hopeless case, shows signs of improvement. I continue the ammoniated tincture of quinine and brandy, giving three doses on the second day, taking great care always to keep the eyes and nose as clear of discharge as possible. On the third day, if the animal is much improved and can walk about a little, I then change over to the pills before mentioned. Fourth day continue pills; fifth day, if animal goes on improving, alternate the pills with the following: ℞. Quinine sulph., gr. j; ferri sulph., gr. j; ext. gentian, gr. ij; quassiæ pulv., gr. iv; gacekient, ad. q. s.; ft. pil. i.

I look upon continual feeding with nutritious materials as of the greatest importance. This I do every two hours when the animal is very much prostrated, with milk and eggs or kreochyle, and keeping with this until the dog shows inclination to eat a little of its own accord, which is usually, in bad cases, about the fifth or sixth day. (Not to cause the animal uneasiness during the administration of medicines, etc., I have used for two years or more a special dog drenching-syringe, made to my order by Messrs. Krohne & Sesemann, 8 Duke Street, Manchester Square, upon which all the graduations for doses are made, whereby I can administer to a dog, without help, a pint of milk and eggs at a time, or give any dose of medicine. The syringe holds one and one-half ounces, and is made so that all parts can be cleaned directly after use.) With commencement of return of appetite the discharge from eyes and nose gradually abates after the fifth day, and if the dog commences to eat I give him meat chopped up very fine, also biscuits, and supplement the feeding by giving bovril, beef tea, extract of meat, etc., at the same time leaving off the influenza pills and giving the tonics only as the symptoms abate. If any complications set in I treat accordingly.

When the discharge from the eyes and nose stops and the animal is getting on well, but remains weak, I give Macadam's cod-liver oil and malt-extract, and feed on Macadam's malt-extract, dog-biscuits, meat, etc., until strength is restored.

*Gastric and Gastro-enteric Forms.* I find the following mixture excellent, together with local treatment, fomentations, etc.: ℞. Liq. calcis iodinatæ, ♏v; vinum ipecac, ♏v; aqua, ad. ℥ij. One dose every two or three hours, regulating all doses according to the size of the patient. I endeavor to work upon the bowels by enemas, if costive, and chlorodyne if the reverse. In addition to this constant feeding is required. In bad cases the only food that a dog seems to retain and digest is a preparation known as "kreochyle." This is a most concentrated form of nourishment, containing all the albumen of meat in solution, and can be digested without the aid of the digestive organs. Thus we can obtain for our patients rest of the inflamed parts. It is prepared by the Kreochyle Company, Viaduct House, Farringdon Street, E. C. I give from one to two teaspoonfuls every one, two or three hours. It has also the advantage that the doses are small, and nourishment of the maximum in each dose. When I consider the animal well enough I then go on with the treatment as for the thoracic form. I hope, therefore, that the treatment I have set forth may give to others the success that it has to me in the cure in canine influenza.

In conclusion, I apologize for any errors that may occur in this article in the description of the disease, etc. I have little time at my disposal to write, or I should have sent this article several weeks earlier.—*The Veterinary Record*, August 5, 1899.

